# Deep learning-based predictive biomarker of pathological complete response to neoadjuvant chemotherapy from histological images in breast cancer

**DOI:** 10.1186/s12967-021-03020-z

**Published:** 2021-08-16

**Authors:** Fengling Li, Yongquan Yang, Yani Wei, Ping He, Jie Chen, Zhongxi Zheng, Hong Bu

**Affiliations:** 1 Department of Pathology, West China Hospital, Sichuan University, Chengdu, 610041 China; 2 Institute of Clinical Pathology, West China Hospital, Sichuan University, Chengdu, 610041 China; 3 Department of Head, Neck and Mammary Gland Oncology, Cancer Center, West China Hospital, Sichuan University, Chengdu, 610041 China

**Keywords:** Breast cancer, Neoadjuvant chemotherapy, Deep learning, Digital pathology

## Abstract

**Background:**

Pathological complete response (pCR) is considered a surrogate endpoint for favorable survival in breast cancer patients treated with neoadjuvant chemotherapy (NAC). Predictive biomarkers of treatment response are crucial for guiding treatment decisions. With the hypothesis that histological information on tumor biopsy images could predict NAC response in breast cancer, we proposed a novel deep learning (DL)-based biomarker that predicts pCR from images of hematoxylin and eosin (H&E)-stained tissue and evaluated its predictive performance.

**Methods:**

In total, 540 breast cancer patients receiving standard NAC were enrolled. Based on H&E-stained images, DL methods were employed to automatically identify tumor epithelium and predict pCR by scoring the identified tumor epithelium to produce a histopathological biomarker, the pCR-score. The predictive performance of the pCR-score was assessed and compared with that of conventional biomarkers including stromal tumor-infiltrating lymphocytes (sTILs) and subtype.

**Results:**

The pCR-score derived from H&E staining achieved an area under the curve (AUC) of 0.847 in predicting pCR directly, and achieved accuracy, F1 score, and AUC of 0.853, 0.503, and 0.822 processed by the logistic regression method, respectively, higher than either sTILs or subtype; a prediction model of pCR constructed by integrating sTILs, subtype and pCR-score yielded a mean AUC of 0.890, outperforming the baseline sTIL-subtype model by 0.051 (0.839, *P * =  0.001).

**Conclusion:**

The DL-based pCR-score from histological images is predictive of pCR better than sTILs and subtype, and holds the great potentials for a more accurate stratification of patients for NAC.

**Supplementary Information:**

The online version contains supplementary material available at 10.1186/s12967-021-03020-z.

## Background

Neoadjuvant chemotherapy (NAC) has been widely used as a standard treatment for patients with locally advanced and sometimes large operable breast cancers [1]. As reported in previous studies [2, 3], NAC not only facilitates the reduction of the tumor burden and increases the rate of breast preservation but also enables the assessment of sensitivity to different treatment regimens in vivo. Correspondingly, the assessment of treatment response to NAC requires a pathological examination. Patients who have the pathological complete response (pCR) are expected to have a better outcome than those with the pathological noncomplete response (non-pCR) [4]. Therefore, pCR after NAC has been regarded as a surrogate endpoint of favorable survival for NAC [5]. However, NAC is not effective for all patients, and only a subset of them can achieve pCR [6]. Patients who do not achieve pCR may suffer from toxic effects during NAC, which is likely to worsen their prognosis while accruing high treatment costs. Therefore, predicting pCR before NAC of breast cancers has significant value in sparing the patients from possibly ineffective treatment.

At present, the biomarkers of tumor size [7], histological grade [8], Ki67 [9], immunochemistry (IHC)-based subtype [8, 10, 11], and stromal tumor-infiltrating lymphocytes (sTILs) [12–14] are the clinicopathological (CP) factors used in predicting pCR partly due to their wide availability in routine clinical practice. Briefly, small tumor size [7], high grade [8], high Ki67 expression [9]], and high sTILs [12–14] are positively related to pCR in NAC settings, while hormone receptor-positive (HR +) or human epidermal growth factor receptor 2-negative (HER2 −) subtypes have lower pCR rates than HR- or HER2 + subtypes [10, 11]. However, these easy-to-get factors from CP data are not robust enough for pCR prediction in all breast cancer patients. In the meantime, although recent studies have proposed some molecular signatures to predict pCR to NAC in breast cancer [15–18], those have been validated in only some of the conducted trials, and they also have the substantial drawbacks of high cost and a considerable time investment. Therefore, there is still an urgent need to develop robust and inexpensive biomarkers for the prediction of pCR to NAC in breast cancer.

Apart from the CP and molecular biomarkers for pCR prediction, artificial intelligence technologies applied in image data can develop predictive signatures by extracting hidden information directly from medical images, such as applications in radiological images involving diffuse optical spectroscopy [19], MRI [20], and PET/CT [21], to predict the treatment response to NAC in breast cancer. Compared with radiological images, histological images, which have been regarded as the gold standard for disease diagnosis, can provide more abundant information on tumor characteristics reflecting underlying molecular processes and disease progression. However, the complex and abundant information from histological images is difficult to adequately use because human assessment mainly relies on visually visible features, while deep learning (DL) technology can address the problem by integrating the visible and subvisible information of recurring patterns from complex images [22]. For instance, the cooperation of DL technology and histological images has shown positive results in processing clinical matters, such as tumor detection [23] and prognosis prediction [24, 25], even presenting satisfying performance in complex matters such as the prediction of gene mutations [26], classification of multimolecular profiles [27], and microsatellite instability [28] among different cancer types. Additionally, a recent investigation in rectal cancer reported that quantitative features extracted by machine learning from histological images can be predictive for treatment response to neoadjuvant chemoradiotherapy (NCRT) [29]. According to these studies, we argue that analyzing histological images through DL technology could contribute to developing predictive biomarkers of treatment response to NAC in breast cancer.

In this study, we aimed to develop a DL-based biomarker using H&E-stained images, pCR-score, to predict pCR of breast cancer patients receiving NAC, which presents a stronger prediction ability than the conventional pathological factors of subtype and sTILs.

## Methods

### Study population and slides

A total of 540 patients who received NAC in January 2008 and June 2020 at West China Hospital were retrospectively enrolled. The inclusion criteria were as follows: (1) patients diagnosed with primary breast invasive ductal cancer (IDC) without metastasis via a needle biopsy before NAC; (2) patients receiving NAC regimens based on anthracycline, taxane, or anthracycline combined with taxane (≥ 4 cycles) and not undergoing prior therapy (detailed NAC regimens in Additional file 1: Table S1); and (3) patients underwent surgery after NAC and was confirmed by pathologic examination whether pCR. The exclusion criteria were as follows: (1) patients received a nonstandard treatment regimen, mainly referring to the treatment of HER2 + breast cancers without trastuzumab; (2) patients lacking complete CP data; (3) patients diagnosed with bilateral, multifocal, or special invasive breast cancer; and (4) the pathological slides from the patient’s biopsy were lost, or the H&E-stained slides were of insufficient quality. The process of patient inclusion is summarized in Additional file 1: Figure S1.

Apart from the H&E-stained-slides corresponding to patients enrolled, an additional dataset of 25 H&E-stained IDC slides were designated for developing the automated workflows for tumor epithelium identification.

### Pathological evaluation and data collection

Pretreatment breast biopsies were performed via ultrasound-guided core needle, routinely fixed in 10% neutral buffered formalin, and stained as H&E slides for diagnosis after paraffin embedding. The surgical specimens after NAC were sampled adequately in the form of tissue slides and examined microscopically by experienced pathologists. pCR was defined as ypT0/isN0 (no residual invasive disease in breast and node) (4). The estrogen receptor (ER), progesterone receptor (PR), HER2 status, and the Ki67 index were assessed through IHC. ER/PR positivity was defined as positive nuclei staining no less than 1% of tumor cells [30]. Regarding Ki67 index, samples were divided into a low-expression set (≤ 20%) and a high-expression set (> 20%)[31]. HER2 status was defined as positive only when IHC (3 +) and (or) amplified by fluorescence in situ hybridization (FISH), while breast cancer with IHC (0/1 +) and (or) unamplified by FISH was considered as HER2-negative disease [32]. sTILs were evaluated on H&E-stained slides according to the international recommended guidelines [33], with intervals of 10% from 1 to 90%, separated into low sTILs (< 10%), moderate sTILs (10%  ≤  and  < 40%), and high sTILs(≤ 40%). The nuclear grade was assessed based on the Nottingham grading system, and the presence or absence of necrosis was assessed on diagnostic H&E-stained slides. To reduce the subjectivity of pathological evaluation, examinations of sTILs, nuclear grade, and treatment response were performed by two observers separately, and samples that were scored inconsistently by the two observers were assessed repeatedly until a consensus was reached.

Apart from the factors above, other clinical data including the age of the patient at diagnosis, tumor/node (T/N) stages, and menstrual status were collected at the same time.

### Image processing and model construction

The H&E-stained slides of pretreatment biopsies were scanned at 40 ×  magnification via a Hamamatsu scanner to prepare whole slide images (WSIs) for experiments. Tumor epithelium (TE) regions of WSIs were identified using a DL-based classification approach (details in Additional file 1: Figure S2). Based on a convolutional neural network I (CNN I), a training dataset of 20 WSIs from an additional dataset was used to develop an automated TE identifying model, and the remaining 5 WSIs were used as the test set. Under manual review, TE was annotated inside two representative tumor regions in the training dataset and global image annotations were conducted in the testing dataset as the gold standard to test the performance of CNN I. Besides, the NDP Viewer 2 was applied in the annotation. Tiles identified as tumor epithelium by CNN I were delivered to a convolutional neural network II (CNN II), scoring the probability of the pCR for each tile.

**Fig. 1:**
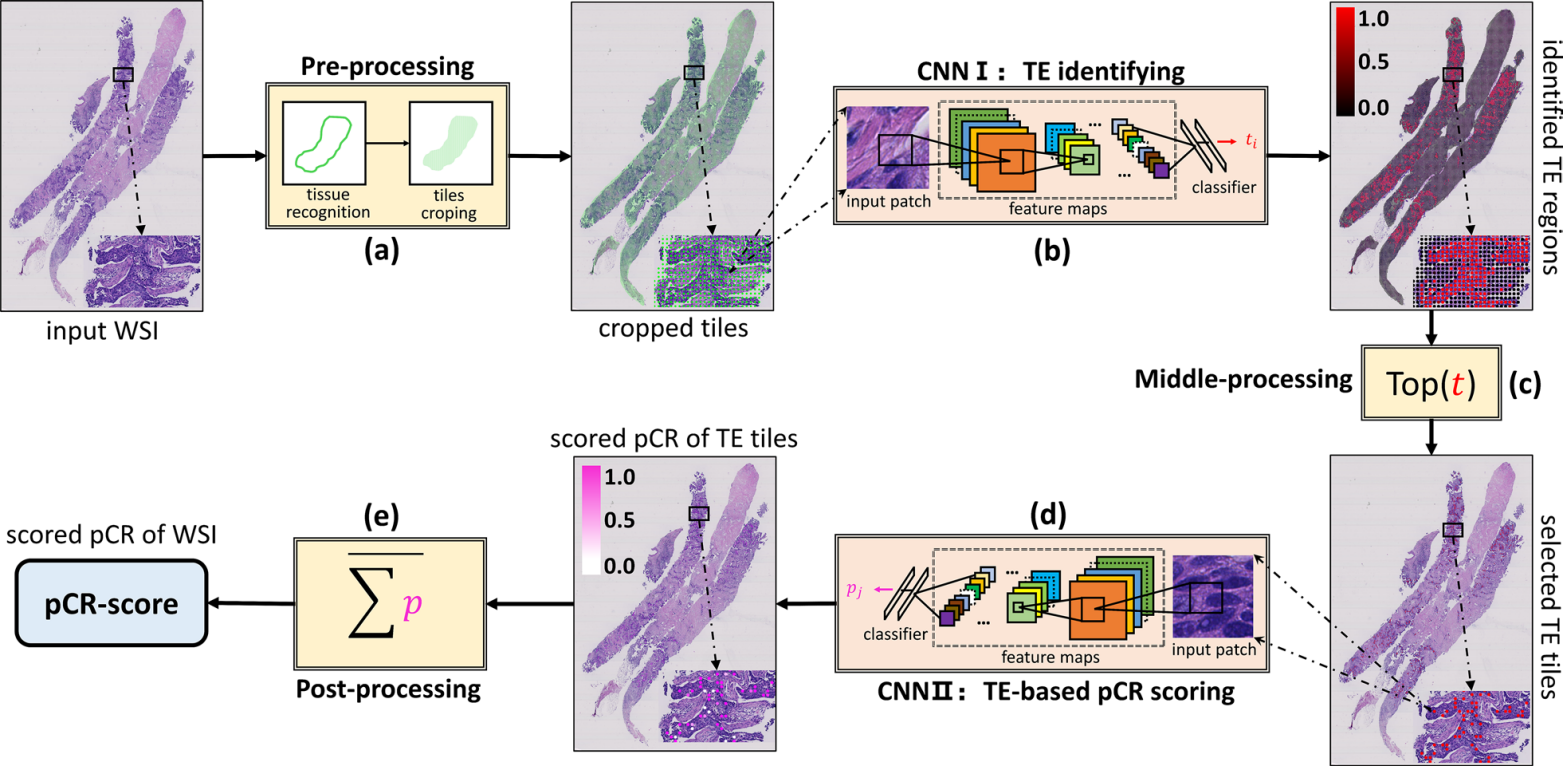
The pipeline of the pCR-score computing consists of five sub-steps: **a** pre-processing; **b** CNN I; **c** middle-processing; **d** CNN II; and **e** post-processing. First, the pre-processing step segments valid tissue areas from the input WSI and crops the segmented valid tissue areas into small tiles. Second, the CNN I takes the cropped tiles as inputs and identifies TE regions by mapping the input tiles into probabilities corresponding to TE. Third, the middle-processing step selects TE tiles with identified high probabilities from the outputs of CNN I. Fourth, the CNN II takes the selected TE tiles as inputs and score the pCR of the input tiles by mapping them into probabilities corresponding to pCR. Finally, the post-processing step fuses the pCR probabilities of TE tiles scored via CNN II to produce the final predicted pCR-score of the input WSI

In the pre-processing step, the developed tissue recognition tool in our previous study [34] was employed to segment the valid tissue areas from the input WSIs, which are cropped into tiles at a scale of 128 × 128 pixels. The deep learning method was employed to automatically identify tumor epithelium and make predictions of pCR based on H&E stained images. CNN I and CNN II were developed based on deep learning methods; Inception V3 was selected as the base deep learning architecture for the presented biomarker generating pipeline, because of its trade off between inferencing speed and classification accuracy [35]. Cross-entropy loss [36] and stochastic gradient descent (SGD) [37] were used in optimization. However, due to that TE tiles were more homogeneous to some extent than original mixed tiles after identified by CNN I, scoring the pCR of these selected TE tiles is more difficult than identifying TE regions in the segmented valid tissue areas. Hence, we first leveraged the recently proposed supervised contrastive learning [38] to optimize the feature extraction part of CNN II to produce features that can distinguish between pCR and non-pCR in the selected tiles. Then, based on the learned discriminative features, we optimized the prediction (classification) part of CNN II by using cross-entropy loss [36] and SGD [37]. Additionally, we leveraged a recently proposed fast ensemble deep learning strategy [39–41] to further boost the optimized CNN II. In the post-processing step, a pCR-score was calculated by averaging the pCR probabilities of TE tiles for each WSI, which was regarded as a novel biomarker for pCR prediction from histology (Fig. 1). More details about the training and inference procedures of CNN I and CNN II are provided in Additional file 1: Figures S2, S4. The whole pipeline of pCR-score computing was implemented using Python based on TensorFlow/Keras.

### Statistic analysis

The distribution of clinical characteristics between cohorts was compared using the χ^2^ test or Fisher’s exact test. The performance of the CNNs for identifying TE and predicting pCR was assessed by the area under the curve (AUC) for the receiver operating characteristic (ROC) curve. Univariate logistic regression analysis was used to evaluate the odds ratios and probabilities of both conventional predictors and pCR-score in correlation with pCR, after which multivariate logistic regression analysis was performed. The Mann–Whitney U test was used to compare the distribution of pCR-scores across patients with different sTILs densities and subtypes. Based on the logistic regression method, the prediction performance of pCR across biomarker-based models was assessed using the F1 score, accuracy, and AUC, along with sensitivity (equal to recall score)/specificity and positive predictive value (PPV, equal to precision)/negative predictive value (NPV), and comparisons of performance metrics among models were performed with the Wilcoxon signed-rank test or a paired t-test as appropriate. Additionally, the pCR-scores were normalized by z score. All statistical analysis was two-sided and *P*-value is less than 0.05 indicating statistical significance. The statistical analyses were performed using SPSS software, version 20.

## Results

### Study population characteristics

According to the inclusion and exclusion criteria, a total of 540 eligible patients were enrolled in this study. Patients were randomly divided into the primary and validation datasets at a ratio of 8:2. The pCR rates were 18.7% in the primary dataset and 19.6% in the validation dataset, and no significant difference was detected in CP factors between the two datasets (Additional file 1: Table S2). The characteristics of the pCR cohort and non-pCR cohort in the two datasets are summarized in Table 1. It was observed that the statuses of ER (*P * <  0.001, *P * <  0.001), PR (*P * <  0.001, *P * =  0.005), and HER2 (*P * <  0.001, *P * <  0.001) were significantly associated with pCR in the primary and validation datasets. Similarly, sTILs at different levels (low, moderate, and high) showed a different distribution between pCR and non-pCR patients in both the primary and validation datasets (*P * <  0.001, *P * <  0.001). However, no significant difference was detected in terms of age, menopausal status, T stage, N stage, or necrosis in pCR and non-pCR cohorts, while the Ki67 index and nuclear grade were significantly correlated with pCR only in the primary dataset but not in the validation dataset.

**Table 1:** Characteristics in the primary and validation datasets

Factors	Primary dataset		*P*	Validation dataset		*P*
	pCR (n = 81)	Non-pCR (n = 352)		pCR (n = 21)	Non-pCR (n = 86)	
Age at diagnosis (%)						
< 50	46 (56.8)	209 (59.4)	0.708	11 (52.4)	48 (55.8)	0.811
≥ 50	35 (43.2)	143 (40.6)		10 (47.6)	38 (44.2)	
Menopausal status (%)						
Premenopausal	45 (55.6)	221 (62.8)	0.255	10 (47.6)	49 (56.9)	0.472
Postmenopausal	36 (44.4)	131 (37.2)		11 (52.4)	37 (43.1)	
T stage (%)						
T1–T2	39 (48.1)	161 (45.7)	0.712	11 (52.4)	39 (45.3)	0.630
T3–T4	42 (51.9)	191 (54.3)		10 (47.6)	47 (54.7)	
N stage (%)						
N0	8 (9.9)	32 (9.1)	1.000	0 (0.0)	5 (5.8)	1.000
N1–N3	73 (90.1)	320 (90.9)		21 (100.0)	81 (94.2)	
ER status (%)						
Positive	26 (32.1)	243 (69.0)	**< 0.001**	4 (19.0)	66 (76.7)	**< 0.001**
Negative	55 (67.9)	109 (31.0)		17 (81.0)	20 (23.3)	
PR status (%)						
Positive	27 (33.3)	231 (65.6)	**< 0.001**	7 (33.3)	59 (68.6)	**0.005**
Negative	54 (66.7)	121 (34.4)		14(66.7)	27 (31.4)	
HER2 status (%)						
Positive	44 (54.3)	75 (21.3)	**< 0.001**	15 (71.4)	17 (19.8)	**< 0.001**
Negative	37 (45.7)	277 (78.7)		6 (28.6)	69 (80.2)	
Subtype (%)						
HR + /HER2 −	16 (19.8)	223 (63.4)	**< 0.001**	3 (14.3)	59 (68.6)	**< 0.001**
HR + /HER2 +	20 (24.7)	40 (11.4)		5 (23.8)	11 (12.8)	
HR −/HER2 −	21 (25.9)	54 (15.3)		3 (14.3)	10 (11.6)	
HR −/HER2 +	24 (29.6)	35 (9.9)		10 (47.6)	6 (7.0)	
Ki-67 index (%)						
≥ 20%	76 (93.8)	296 (84.1)	**0.021**	20 (95.2)	70 (81.4)	0.184
< 20%	5 (6.2)	56 (15.9)		1 (4.8)	16 (18.6)	
Nuclear grade (%)						
1 or 2	40 (49.4)	252 (71.6)	**0.001**	11 (52.4)	59 (68.6)	0.202
3	41 (51.6)	100 (28.4)		10 (47.6)	27 (31.4)	
sTILs (%)						
Low	31 (38.3)	207 (58.8)	**0.001**	4 (19.0)	57 (66.3)	**0.001**
Moderate	44 (54.3)	136 (38.6)		14 (66.7)	26 (30.2)	
High	6 (7.4)	9 (2.6)		3 (14.3)	3 (3.5)	
Necrosis (%)						
Yes	22 (27.2)	67 (19.0)	0.126	6 (28.6)	13 (14.9)	0.200
No	59 (72.8)	285 (81.0)		15 (71.4)	74 (85.1)	

Bold indicates statistical significance (*P * <  0.05)

*ER* estrogen receptor; *PR* progesterone receptor; *HER2* human epidermal growth factor receptor 2; *HR* hormone receptor; *sTILs* stromal tumor-infiltrating lymphocytes; *pCR* pathological complete response

### The pCR-score derived from H&E-stained images is predictive of pCR

In the pre-processing step, an intelligent tool [34] developed previously was employed to segment the valid tissue for the WSI, with tiles of 128 × 128 pixels were generated. Then 44,348 tiles were generated from the training set (20 WSIs), and 20,313 tiles were generated from the test set (5 WSIs), which were used for developing the automated workflows for tumor epithelium identification. CNN I, which subdivided the tiles into TE, and non-TE, achieved an AUC of 0.851 for identifying TE tiles compared with the reference standard (Additional file 1: Figure S3), and other relevant performance metrics were shown in Additional file 1: Table S3.

**Fig. 2:**
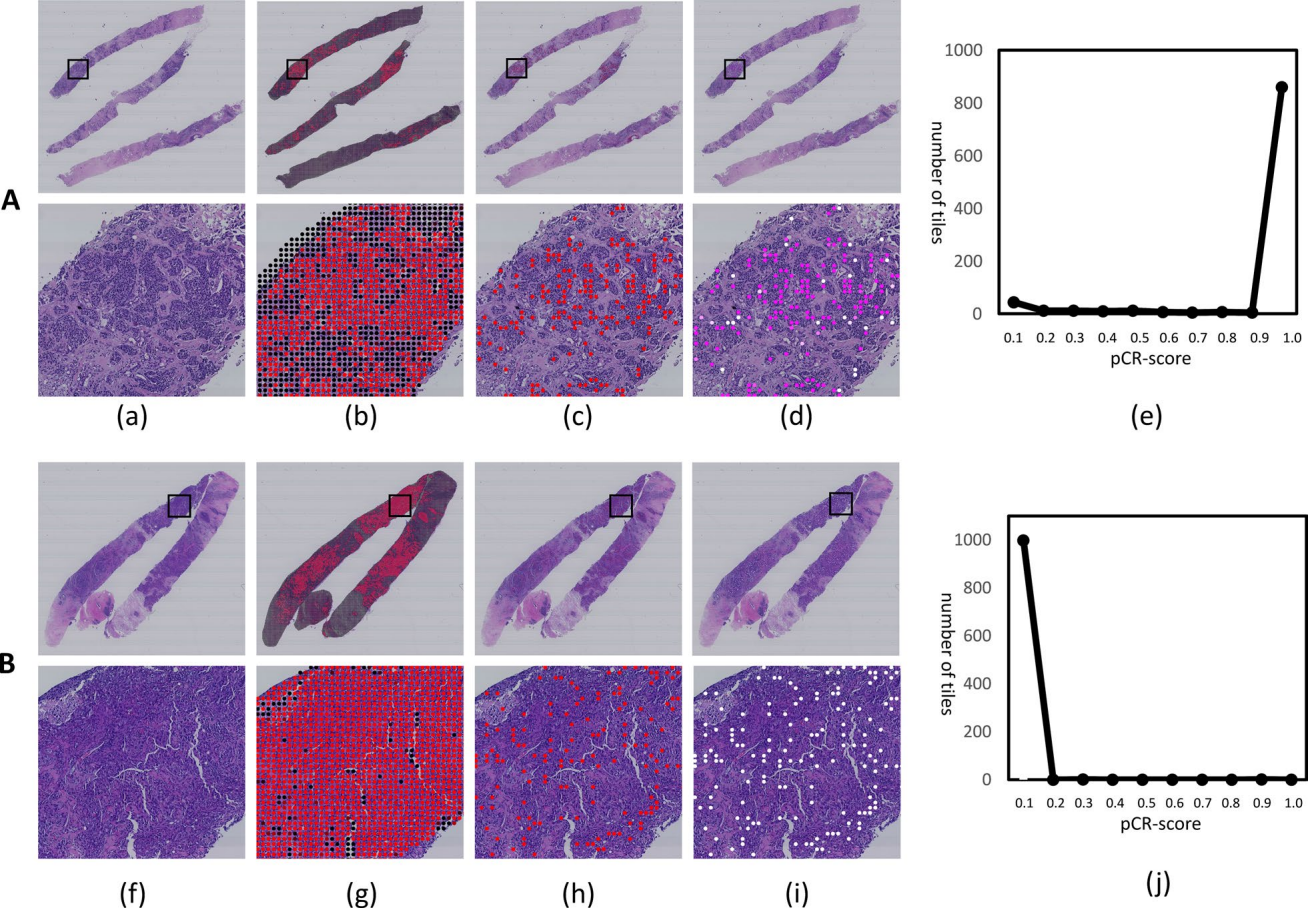
Examples for the pCR prediction of based on CNNs. **A** An example of pCR: **a** shows a represnetative WSI from a patient who achieved the pCR. **b**The probability map of TE produced by the CNN I. **c** The selection of tiles with a high probability of TE. **d** The map produced by CNN II showing the pCR probability for image **a**, where pink and white separately represent high and low probabilities of pCR. **e** The distributions of tile-level pCR scores of the image **a**. **B** An example of non-pCR: **f** shows a representative WSI from a patient who did not achieve the pCR. **g**, **h**, and **i** for image **f** are corresponding to the steps of **b**, **c**, and **d** for image **a**. **j** A predominant distribution of low pCR scores for image **f**

**Fig. 3:**
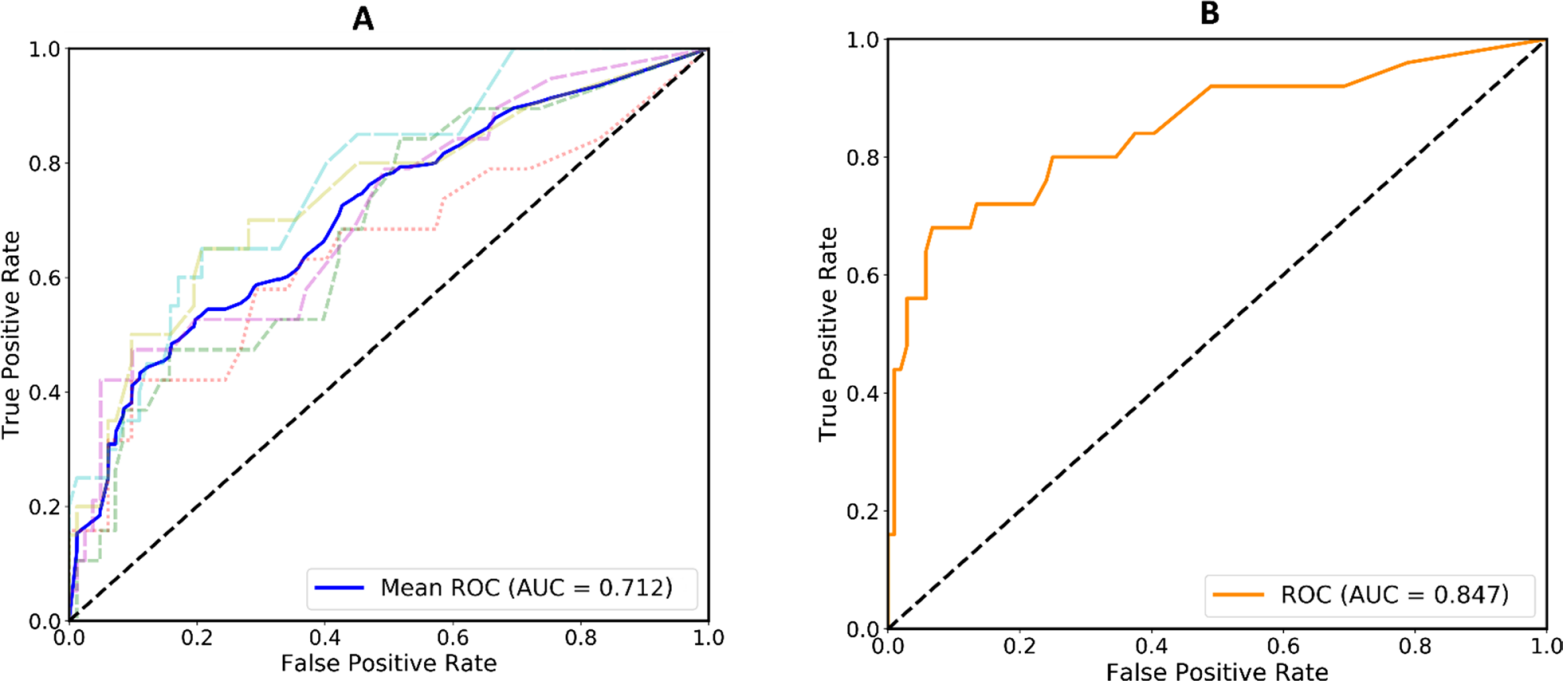
ROC curves of raw pCR-scores based on CNN II for pCR prediction in the primary dataset (**A**) and validation dataset (**B**)

From CNN I,  ≤  1000 TE tiles with identified high probabilities (> 0.9999) per WSI were selected for pCR scoring, resulting in a total of 292,025 tiles for the whole cohort. TE tiles with definite labels of pCR or non-pCR in the primary dataset were used to train CNN II to calculate the probability of pCR for each tile, and the mean risk of all selected tiles was computed as a pCR-score for one WSI (Fig. 1). Examples of DL-based pCR-score generation for patients with pCR and non-pCR are shown in Fig. 2. The five-fold cross-validation on the primary dataset and one test on the validation dataset were conducted to assess performance in predicting pCR to NAC directly using the raw pCR-score data. As shown in Fig. 3, the mean AUC in the primary dataset was 0.712 on the WSI level, while it was 0.847 in the validation dataset. Besides, AUCs at the tile level are provided in Additional file 1: Figure S5. Moreover, the predictive performance of the pCR-score for different subtypes (HR + /HER2 −, HR + /HER2 + , HR −/HER2 − and HR −/HER2 +) on the tile-level and WSI-level are shown in Additional file 1: Figure S6. Distributions of the generated pCR-score in the pCR group and the non-pCR group of the validation dataset are provided in Additional file 1: Figure S7.

### The pCR-score is an independent biomarker correlated with pCR

To evaluate the clinical significance of the pCR-score, univariate and multivariate logistic regression analyses were performed in the validation dataset, including the biomarkers of routine clinical use as well (Table 2). Notably, as CNN II for pCR scoring was built on the primary dataset, the following experiments were conducted only in the validation dataset to avoid overfitting of the pCR-score. In univariate analysis, the pCR-score was a significant biomarker related to pCR with an odds ratio of 3.516 (95% CI 2.003–6.173, *P * <  0.001). Apart from pCR-score, subtype and sTILs were significantly correlated with pCR in the validation dataset, but T stage, Ki67, and nuclear grade were not. Besides, subtype and sTILs were independent markers correlated with pCR in the multivariate analysis without pCR-score; while adding it, pCR-score was the only significant predictor with an odds ratio of 4.045 (95% CI 1.822–8.980, *P * =  0.001). These results showed that the pCR-score was an independent biomarker correlated with pCR.

**Table 2:** Univariate and multivariate analysis for pCR-score and important factors

Factors	Univariate analysis		Multivariate analysis^a^		Multivariate analysis^b^	
	OR (95% CI)	*P*	OR (95% CI)	*P*	OR (95% CI)	*P*
pCR-score	3.516 (2.003–6.173)	**< 0.001**	–	–	4.045 (1.822–8.980)	**0.001**
Subtypes	–	**< 0.001**	–	**0.003**	–	0.084
HR +/HER2-	1	1	1	1	1	1
HR +/HER2 +	8.939 (1.861–42.944)	**0.006**	8.049 (1.484–43.664)	**0.016**	2.899 (0.395–21.282)	0.295
HR-/HER2 −	5.900 (1.041–33.477)	**0.045**	4.798 (0.733–31.387)	0.102	2.327 (0.262–20.671)	0.449
HR-/HER2 +	32.778 (7.031–152.815)	**< 0.001**	21.497 (4.089–113.00)	**< 0.001**	12.923 (1.783–93.638)	**0.011**
sTILs density	–	**0.002**	–	**0.041**	–	0.060
Low	1	1	1	1	1	1
Moderate	7.673 (2.302–25.581)	**0.001**	5.374 (1.311–22.021)	**0.019**	8.256 (1.404–48.546)	**0.020**
High	14.250 (2.143–94.741)	**0.006**	9.206 (0.939–90.247)	0.057	7.379 (0.511–106.471)	0.142
T stage	0.754 (0.290–1.962)	0.563	1.515 (0.428–5.356)	0.519	2.219 (0.487–10.119)	0.303
Ki67	4.571 (0.571–36.610)	0.152	1.166 (0.116–11.737)	0.897	1.859 (0.142–24.352)	0.636
Nuclear grade	1.987 (0.753–5.240)	0.165	1.024 (0.283–3.708)	0.971	1.076 (0.233–4.956)	0.926

^a^Multivariate analysis excluding the pCR-score

^b^Multivariate analysis including the pCR-score

### The pCR-score outperforms biomarkers of sTILs and subtype in predicting pCR

**Fig. 4:**
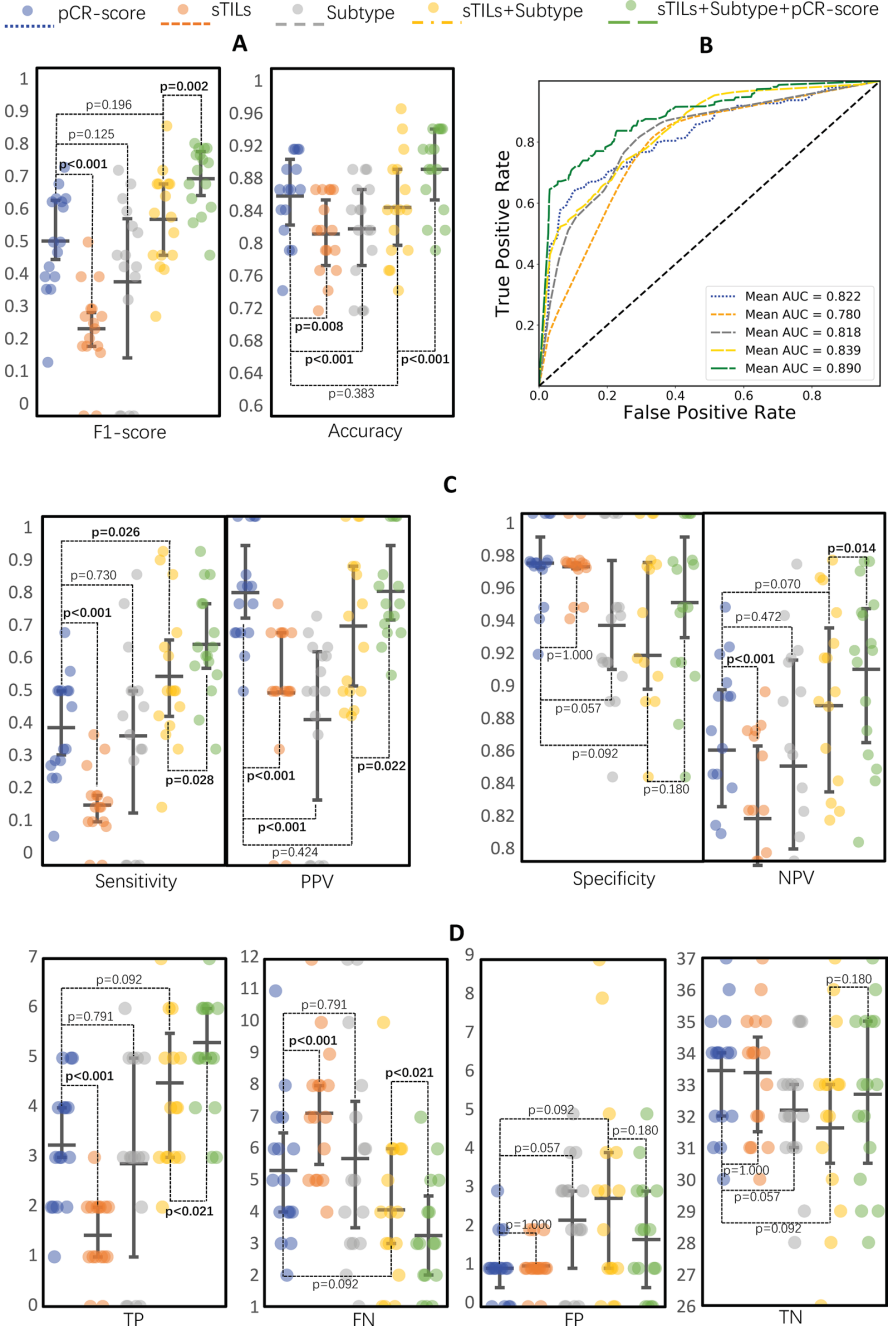
Comparisons of the pCR prediction performance metrics of sTILs, subtype, and pCR-score in the 16-time repeated validation (In each repeat, we randomly select 60% of the data as training data, and the remaining 40% as testing data. Mean values of each metric were calculated from the 16 repeats to avoid the impact of data bias). **A** Comparisons of the F1 score and accuracy of models. **B** Comparisons of the AUCs of models. **C** Comparisons of the sensitivity (equal to recall score), PPV (equal to precision score), specificity, and NPV of models. **D** Comparisons of TP, FN, FP, and TN in confusion matrices among models

For comparisons with pCR-score, we also assessed the predictive ability of the baseline biomarkers using logistic regression models. 60% WSIs of the validation dataset were randomly selected as the training set to build prediction models with biomarkers and the remaining 40% were taken as the test set to compare the performance. This procedure was repeated 16 times to avoid unfair assessments due to data bias (Fig. 4; Table 3; and Additional file 1: Table S4). The prediction model based on the pCR-score presented better performance in predicting pCR than sTILs/subtype-based model, especially showed significantly higher accuracy and PPV/precision (Table 3: 0.853 vs. 0.810/0.815, *P * <  0.001, *P * =  0.008; 0.781 vs.0.494/0.418, *P * <  0.001, *P * <  0.001), even rivaling that of a baseline model combined sTILs and subtype (Table 3). Other detailed performance metrics are available in Fig. 4; Table 3. Moreover, the T stage/Ki67/nuclear grade-based models showed poor predictive ability, and the addition of them did not improve the baseline model (Table 3; Additional file 1: Table S4).

**Table 3:** Performance metrics of biomarker-based models

Metrics	pCR-score-based		sTILs-based^a^		Subtype-based^a^		Baseline^a^		Integrated^b^	
	Mean (95% CI)	*P*	Mean (95% CI)	*P*	Mean (95% CI)	*P*	Mean (95% CI)	*P*	Mean (95% CI)	*P*
F1 score	0.503 (0.424–0.581)	–	0.250 (0.181–0.320)	**< 0.001**	0.386 (0.252–0.519)	0.100	0.565 (0.491–0.639)	0.196	0.682 (0.631–0.732)	**0.002**
Accuracy	0.853 (0.827–0.879)	–	0.810 (0.786–0.834)	**< 0.001**	0.815 (0.785–0.846)	**0.008**	0.840 (0.808–0.873)	0.380	0.884 (0.859–0.909)	**< 0.001**
AUC	0.822 (0.784–0.861)	–	0.780 (0.753–0.806)	**< 0.001**	0.818 (0.782–0.855)	0.858	0.839 (0.812–0.866)	0.485	0.890 (0.863–0.916)	**0.001**
Sensitivity	0.396 (0.314–0.474)	–	0.173 (0.118–0.226)	**< 0.001**	0.370 (0.230–0.511)	0.730	0.541 (0.433–0.648)	**0.026**	0.633 (0.552–0.715)	**< 0.001**
PPV	0.781 (0.699–0.864)	–	0.494 (0.377–0.612)	**< 0.001**	0.418 (0.278–0.559)	**< 0.001**	0.686 (0.571–0.801)	0.424	0.785 (0.707–0.863)	**0.022**
Specificity	0.971 (0.959–0.984)	–	0.969 (0.961–0.978)	1.000	0.936 (0.912–0.960)	0.057	0.918 (0.876–0.961)	0.092	0.945 (0.924–0.973)	0.180
NPV	0.864 (0.835–0.893)	–	0.823 (0.798–0.851)	**< 0.001**	0.855 (0.815–0.895)	0.472	0.889 (0.857–0.922)	0.070	0.910 (0.884–0.936)	0.144
TP	3.250 (2.590–3.910)	–	1.438 (1.004–1.871)	**< 0.001**	2.875 (1.794–3.956)	0.791	4.500 (3.527–5.473)	0.092	5.313 (4.517–6.108)	**0.021**
FN	5.313 (4.118–6.508)	–	7.125 (5.993–8.258)	**< 0.001**	5.688 (3.897–7.478)	0.791	4.063 (2.780–5.345)	0.092	3.250 (2.287–4.213)	**0.021**
FP	1.000 (0.565–1.435)	–	1.063 (0.757–1.368)	1.000	2.250 (1.413–3.087)	0.057	2.813 (1.367–4.258)	0.092	1.750 (0.936–2.564)	0.180
TN	33.44 (32.41–34.47)	–	33.38 (32.29–34.46)	1.000	32.19 (31.14–33.29)	0.057	31.63 (29.84–33.41)	0.092	32.69 (31.24–34.14)	0.180

Bold indicates statistical significance (*P * <  0.05)

*pCR* pathological complete response; *sTILs* stromal tumor-infiltrating lymphocytes; *OR* odds ratio; *CI* confidence interval; *AUC* area under the curve

^a^*P* value was from that the comparisons with pCRscore-based model

^b^solely refers to the *P* value was from the comparisons of baseline model and integrated model (baseline: sTILs  +  subtype; integrated: sTILs  +  subtype  +  pCR-score)

Moreover, an integrated logistic regression model was constructed based on the biomarkers of sTILs, subtype, and pCR-score to assess the relative ability of the pCR-score to predict pCR in comparison with the baseline model. With the addition of the pCR-score in the integrated model, we found that the mean performance metrics were significantly improved from 0.840 to 0.884 (*P * <  0.001), 0.839 to 0.890 (*P * =  0.001), and 0.565 to 0.682 (*P * =  0.002) in the accuracy, AUC, and F1 score respectively, and other metrics including sensitivity/recall, PPV/precision were significantly improved while specificity and NPV were increased without significance (detailed metrics in Fig. 4; Table 3).

### Distributions of the pCR-score varied among subtypes

**Fig. 5:**
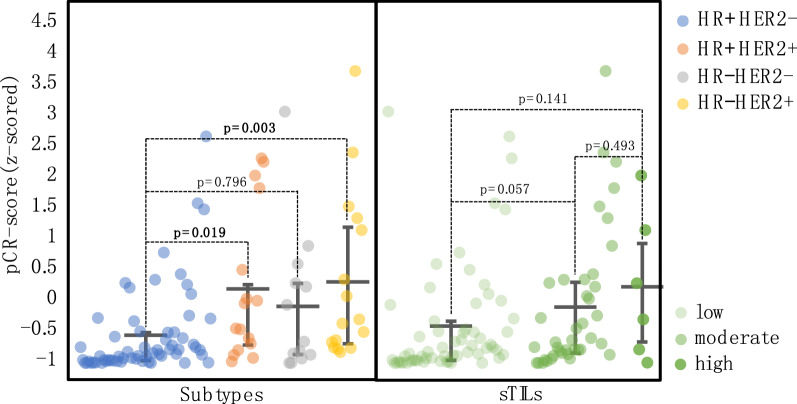
The distributions of pCR-scores across different subtypes and sTILs densities

To further investigate the relationship of the pCR-score with sTILs and subtype, we visualized how the pCR-score was distributed across patients of different subtypes and sTILs densities (Fig. 5). Patients with the HR −/HER2 + or HR + /HER2 + subtypes appeared to have higher pCR-scores than those with the HR + /HER2 − subtype (*P* = 0.003, *P* = 0.019). However, HR −/HER2 − had an intermediate pCR-score among the four subtypes, showing a slightly higher trend of pCR-score than that of the HR + /HER2 − subtype without significance (*P * =  0.79). Additionally, we visualized the distribution of the pCR-score in different sTILs densities. A trend toward elevated pCR-scores was observed in patients with higher sTIL density, but the difference between the distributions was not significant (Fig. 5).

## Discussion

In this study, we proposed the DL-based pCR-score, probably the first biomarker without predefined features from H&E-stained slides, which indicated the predictive potentials of the histological images for treatment response. The pCR-score presented herein is an independent predictor correlating with pCR in multivariate analysis, and it outperforms the conventional biomarkers in predicting pCR. Moreover, further experiments show that the pCR-score reflects additional predictive information solely from H&E-stained slides and is complementary to the existing biomarkers, providing a more robust prediction of pCR to identify the patients who are most likely to benefit from NAC.

Breast cancer is characterized by high heterogeneity of morphology reflecting the underlying molecular process, which can provide indicative information for clinical decision-making. For instance, nuclear pleomorphism is an essential constituent of the breast histological grading system, which implies the aggressiveness of the disease and is related to prognosis. Apart from the manual assessment of the morphological features, computer-extracted features of morphology like the nuclear shape/texture were capable of predicting the patient survival [42]. These facts proved that the morphological characteristics of breast cancer could provide essential information on the disease. In this study, our results showed that the DL-based raw pCR-scores derived solely from H&E-stained slides achieved an AUC of 0.847 in predicting pCR; this simple predictor is not based on prior knowledge of breast biology or pathology, which implies that histological images contain potential information predicting the treatment response of breast cancer.

In one study of 58 breast cancer patients, Dodington et al*. *[43] focused on the nuclear level after segmentation was used to extract a limited set of nuclear features for analyses; the nuclear intensity and gray-level co-occurrence matrix (GLCM-COR) of tumor nuclear features were found to be related to pCR in univariate analysis (*P* = 0.035, *P * =  0.039). Differently, our learning process with CNN II was guided simply by the assessment results of the treatment response instead of focusing on specific features of tumor nuclear morphology, which allowed us to explore a wider range of image information values. During the image-processing step, we set up automated workflows to identify the regions of interest (ROIs), including valid tissue detection [34] as the first step followed by TE identification based on CNN I, which addressed the problem of manual annotations for large image datasets by automating the annotation process. Notably, we found that there was a smaller mean number of TE tiles in patients with pCR who were not correctly predicted than in patients with pCR who were correctly predicted (603 vs 858), and the prediction accuracy of pCR for patients with  ≥  500 tiles was higher than patients with  <  500 tiles (50% vs 10%), which implied that the small number of tumor tissue could not reflect sufficient morphological information, resulting in an unfair assessment of pCR-score. A possibility is the high heterogeneity of breast cancers, especially in a small quantity of sampled tissue of tumor biopsies, which also can affect the manual evaluation of histopathological features.

At present, information derived from medical images has been accepted as a novel prognostic and predictive biomarker in oncology [22, 24, 44, 45]. For example, Skrede et al. [24] developed a useful DL-based prognostic biomarker from histological images and proved it outperformed established molecular and morphological prognostic markers; Kather et al. [44] proposed the deep stroma score, a DL-based biomarker solely from H&E staining images, which was demonstrated to be an independent prognostic factor in colorectal cancer. In the present study, we proposed the pCR-score, independently correlated with pCR, is a promising biomarker that can classify breast cancer patients as “potential responders” or “potential non-responders” solely based on H&E-stained images. Unlike conventional histopathological biomarkers which require manual assessments, the pCR-score from scanned images is generated by the DL systems without the extra effort of manual evaluation, which prevents high intra-observer subjectivity and inter-observer variation. The DL-based pCR-score can provide complementary information that is not being extracted from routine material in current clinical workflows, whose combination with conventional biomarkers has the potential of better stratifying patients to decide which individuals are more likely to benefit from NAC.

We also found that the distributions of pCR-scores across different subtypes of breast cancers were varied, which were correlated with the response rates of subtypes to NAC [10, 11]. For example, higher pCR-scores are more likely to appear in patients with HER2 + subtypes or HR − subtypes (the lack of a significant difference between the HR −/HER2 − and HR +/HER2 − subtypes might be due to the small number of HR −/HER2 − samples), which suggests that the pCR-score derived from H&E staining images might reflect the histological difference associated with subtypes to predict treatment response to NAC. Indeed, published data support the idea that morphological features of breast cancer can provide the subtype information [46]. However, the differences in the distribution of pCR-scores among sTILs densities were not significant, since the pCR-score is derived from the TE while the assessment of sTILs is focused on stromal regions.

Although our study demonstrated the potential of tumor histology to predict pCR via DL approaches and proposed a novel biomarker that is a more effective predictor than sTILs or subtype, it still has some limitations. In this study, only 540 patients were used retrospectively for training and validation; hence, future studies should pursue prospective multicenter investigations. Second, only some of the tiles from each WSI were used for training and prediction; subsequent research should aim to develop more advanced methods to incorporate more tiles to better account for the heterogeneity of breast cancer. Third, although the pCR-score does not rely on the manual assessment as sTILs and subtype, it needs an expert of data analyst to generate. Additionally, although the integrated model was objectively superior to others, which has already supported our conclusion, its F1 score and sensitivity were not subjectively high; future improvement of it in a large cohort is essentially required for us. Alternatively, considering that this study demonstrated the value of the TE in predicting the treatment response to NAC, we will continue to explore the predictive potential of the nontumor compartment of breast cancer via the DL approaches.

## Conclusion

Conclusively, we proposed the pCR-score, a promising DL-based histological biomarker, and demonstrated its excellent performance in predicting pCR to NAC exceeding the basic biomarkers of sTILs and subtype. The pCR-score facilitates the better stratification of breast cancer patients for NAC; with more DL-based biomarkers developed, a more robust predictive model may be created to assist clinical treatment planning.

## Supplementary Information

**Additional file 1**: **Figure S1.** The workflow of patient selection. **Figure S2.** Learning and inference processes of CNN I for TE identification. **Figure S3.** ROC curve and confusion matrices of CNN I for identifying TE. **Figure S4.** Learning and inference processes of CNN II for pCR prediction. **Figure S5.** ROC curves of CNN II for pCR prediction at tile-level. **Figure S6.** ROC curves of CNN II for pCR prediction based on TE on tile-level and WSI-level among subtypes in validation. **Figure S7.** Distributions of the pCR-score in the pCR group and the non-pCR group of the validation dataset. **Table S1.** Detailed NAC regimens of patients. **Table S2.** Demographic comparison between the primary and validation datasets. **Table S3.** Performance metrics of CNN I including accuracy, F1 score, AUC, sensitivity (recall), PPV (precision), specificity, and NPV. **Table S4.** Performance metrics including sensitivity (recall), PPV (precision), specificity, and NPV for biomarker-based models (T stage, nuclear grade, and Ki67).

## Data Availability

The datasets and related codes used and/or analyzed during the current study are available from the corresponding author on reasonable request.

## Abbreviations

pCR: Pathological complete response
NAC: Neoadjuvant chemotherapy
DL: Deep learning
H&E: Hematoxylin and eosin
AUC: Area under the curve
sTILs: Stromal tumor-infiltrating lymphocytes,
IHC: Immunochemistry
CP: Clinicopathological
HR: Hormone receptor
HER2: Human epidermal growth factor receptor 2
NCRT: Neoadjuvant chemoradiotherapy
IDC: Invasive ductal cancer
ER: Estrogen receptor
PR: Progesterone receptor
FISH: Fluorescence in situ hybridization
WSI: Whole slide image
TE: Tumor epithelium
CNN: Convolutional neural network
SGD: Stochastic gradient descent
ROC: Receiver operating characteristic
PPV: Positive predictive value
NPV: Negative predictive value
GLCM-COR: Gray-level co-occurrence matrix
ROI: Region of interest

## References

[1] Gradishar WJ, Anderson BO, Abraham J, Aft R, Agnese D, Allison KH (2020). Breast cancer, version 3.2020, NCCN clinical practice guidelines in oncology. J Natl Compr Cancer Netw JNCCN.

[2] Derks MGM, van de Velde CJH (2018). Neoadjuvant chemotherapy in breast cancer: more than just downsizing. Lancet Oncol.

[3] von Minckwitz G, Blohmer JU, Costa SD, Denkert C, Eidtmann H, Eiermann W (2013). Response-guided neoadjuvant chemotherapy for breast cancer. J Clin Oncol.

[4] Cortazar P, Zhang L, Untch M, Mehta K, Costantino JP, Wolmark N (2014). Pathological complete response and long-term clinical benefit in breast cancer: the CTNeoBC pooled analysis. Lancet.

[5] Esserman LJ, Woodcock J (2011). Accelerating identification and regulatory approval of investigational cancer drugs. JAMA.

[6] Spring L, Greenup R, Niemierko A, Schapira L, Haddad S, Jimenez R (2017). Pathologic complete response after neoadjuvant chemotherapy and long-term outcomes among young women with breast cancer. J Natl Compr Cancer Netw JNCCN.

[7] Goorts B, van Nijnatten TJ, de Munck L, Moossdorff M, Heuts EM, de Boer M (2017). Clinical tumor stage is the most important predictor of pathological complete response rate after neoadjuvant chemotherapy in breast cancer patients. Breast Cancer Res Treat.

[8] Lips EH, Mulder L, de Ronde JJ, Mandjes IA, Koolen BB, Wessels LF (2013). Breast cancer subtyping by immunohistochemistry and histological grade outperforms breast cancer intrinsic subtypes in predicting neoadjuvant chemotherapy response. Breast Cancer Res Treat.

[9] Alba E, Lluch A, Ribelles N, Anton-Torres A, Sanchez-Rovira P, Albanell J (2016). High proliferation predicts pathological complete response to neoadjuvant chemotherapy in early breast cancer. Oncologist.

[10] Haque W, Verma V, Hatch S, Suzanne Klimberg V, Brian Butler E, Teh BS (2018). Response rates and pathologic complete response by breast cancer molecular subtype following neoadjuvant chemotherapy. Breast Cancer Res Treat.

[11] Houssami N, Macaskill P, von Minckwitz G, Marinovich ML, Mamounas E (2012). Meta-analysis of the association of breast cancer subtype and pathologic complete response to neoadjuvant chemotherapy. Eur J Cancer.

[12] Denkert C, von Minckwitz G, Darb-Esfahani S, Lederer B, Heppner BI, Weber KE (2018). Tumour-infiltrating lymphocytes and prognosis in different subtypes of breast cancer: a pooled analysis of 3771 patients treated with neoadjuvant therapy. Lancet Oncol.

[13] Ali HR, Dariush A, Thomas J, Provenzano E, Dunn J, Hiller L (2017). Lymphocyte density determined by computational pathology validated as a predictor of response to neoadjuvant chemotherapy in breast cancer: secondary analysis of the ARTemis trial. Ann Oncol.

[14] Denkert C, Loibl S, Noske A, Roller M, Muller BM, Komor M (2010). Tumor-associated lymphocytes as an independent predictor of response to neoadjuvant chemotherapy in breast cancer. J Clin Oncol.

[15] Carey LA, Berry DA, Cirrincione CT, Barry WT, Pitcher BN, Harris LN (2016). Molecular heterogeneity and response to neoadjuvant human epidermal growth factor receptor 2 targeting in CALGB 40601, a randomized phase III trial of paclitaxel plus trastuzumab with or without lapatinib. J Clin Oncol.

[16] Abdel-Fatah TMA, Agarwal D, Liu DX, Russell R, Rueda OM, Liu K (2016). SPAG5 as a prognostic biomarker and chemotherapy sensitivity predictor in breast cancer: a retrospective, integrated genomic, transcriptomic, and protein analysis. Lancet Oncol.

[17] Pineda B, Diaz-Lagares A, Pérez-Fidalgo JA, Burgués O, González-Barrallo I, Crujeiras AB (2019). A two-gene epigenetic signature for the prediction of response to neoadjuvant chemotherapy in triple-negative breast cancer patients. Clin Epigenetics.

[18] Alba E, Rueda OM, Lluch A, Albanell J, Chin S-F, Chacon JI (2018). Integrative cluster classification to predict pathological complete response to neoadjuvant chemotherapy in early breast cancer. J Clin Oncol.

[19] Tran WT, Gangeh MJ, Sannachi L, Chin L, Watkins E, Bruni SG (2017). Predicting breast cancer response to neoadjuvant chemotherapy using pretreatment diffuse optical spectroscopic texture analysis. Br J Cancer.

[20] Cain EH, Saha A, Harowicz MR, Marks JR, Marcom PK, Mazurowski MA (2019). Multivariate machine learning models for prediction of pathologic response to neoadjuvant therapy in breast cancer using MRI features: a study using an independent validation set. Breast Cancer Res Treat.

[21] Lee H, Lee DE, Park S, Kim TS, Jung SY, Lee S (2019). Predicting response to neoadjuvant chemotherapy in patients with breast cancer: combined statistical modeling using clinicopathological factors and FDG PET/CT texture parameters. Clin Nucl Med.

[22] Echle A, Rindtorff NT, Brinker TJ, Luedde T, Pearson AT, Kather JN (2020). Deep learning in cancer pathology: a new generation of clinical biomarkers. Br J Cancer.

[23] Ehteshami Bejnordi B, Veta M, van Johannes Diest P, van Ginneken B, Karssemeijer N, Litjens G (2017). Diagnostic assessment of deep learning algorithms for detection of lymph node metastases in women with breast cancer. JAMA.

[24] Skrede OJ, De Raedt S, Kleppe A, Hveem TS, Liestøl K, Maddison J (2020). Deep learning for prediction of colorectal cancer outcome: a discovery and validation study. Lancet.

[25] Mobadersany P, Yousefi S, Amgad M, Gutman DA, Barnholtz-Sloan JS, Velázquez Vega JE (2018). Predicting cancer outcomes from histology and genomics using convolutional networks. Proc Natl Acad Sci USA.

[26] Coudray N, Ocampo PS, Sakellaropoulos T, Narula N, Snuderl M, Fenyö D (2018). Classification and mutation prediction from non-small cell lung cancer histopathology images using deep learning. Nat Med.

[27] Woerl AC, Eckstein M, Geiger J, Wagner DC, Daher T, Stenzel P (2020). Deep learning predicts molecular subtype of muscle-invasive bladder cancer from conventional histopathological slides. Eur Urol.

[28] Kather JN, Pearson AT, Halama N, Jäger D, Krause J, Loosen SH (2019). Deep learning can predict microsatellite instability directly from histology in gastrointestinal cancer. Nat Med.

[29] Zhang F, Yao S, Li Z, Liang C, Zhao K, Huang Y (2020). Predicting treatment response to neoadjuvant chemoradiotherapy in local advanced rectal cancer by biopsy digital pathology image features. Clin Transl Med.

[30] Allison KH, Hammond MEH, Dowsett M, McKernin SE, Carey LA, Fitzgibbons PL (2020). Estrogen and progesterone receptor testing in breast cancer: ASCO/CAP guideline update. J Clin Oncol.

[31] Goldhirsch A, Winer EP, Coates AS, Gelber RD, Piccart-Gebhart M, Thürlimann B (2013). Personalizing the treatment of women with early breast cancer: highlights of the St Gallen International Expert Consensus on the Primary Therapy of Early Breast Cancer 2013. Ann Oncol.

[32] Wolff AC, Hammond MEH, Allison KH, Harvey BE, Mangu PB, Bartlett JMS (2018). Human epidermal growth factor receptor 2 testing in breast cancer: American Society Of Clinical Oncology/College of American Pathologists Clinical Practice Guideline Focused Update. J Clin Oncol.

[33] Salgado R, Denkert C, Demaria S, Sirtaine N, Klauschen F, Pruneri G (2015). The evaluation of tumor-infiltrating lymphocytes (TILs) in breast cancer: recommendations by an International TILs Working Group 2014. Ann Oncol.

[34] Yongquan Y, inventor; Chengdu Gaoyuan Intellectual Property Agency, assignee. Pathological section tissue region recognition system based on image semantic segmentation. China patent 201911204394. 29 Nov 2019.

[35] Szegedy C, Vanhoucke V, Ioffe S, Shlens J, Wojna Z (2016). Rethinking the inception architecture for computer vision. 2016 IEEE conference on computer vision and pattern recognition (CVPR).

[36] Wu YN, Ikeuchi K (2014). Cross entropy. Computer vision: a reference guide.

[37] Theodoridis S, Theodoridis S (2015). Chapter 5—stochastic gradient descent: the LMS algorithm and its family. Machine learning.

[38] Khosla P, Teterwak P, Wang C, Sarna A, Tian Y, Isola P, et al. Supervised contrastive learning. ArXiv. 2020. abs/2004.11362. Accessed 10 Mar 2021.

[39] Yang Y, Lv H, Chen N, Wu Y, Zheng J, Zheng Z (2020). Local minima found in the subparameter space can be effective for ensembles of deep convolutional neural networks. Pattern Recognit.

[40] Yongquan Y, Haijun L, Ning C, Yang W, Zhongxi Z (2020). FTBME: feature transferring based multi-model ensemble. Multimed Tools Appl.

[41] Yang Y, Lv H. Discussion of ensemble learning under the era of deep learning. ArXiv. 2021. abs/2101.08387. Accessed 25 Jan 2021.

[42] Lu C, Romo-Bucheli D, Wang X, Janowczyk A, Ganesan S, Gilmore H (2018). Nuclear shape and orientation features from H&E images predict survival in early-stage estrogen receptor-positive breast cancers. Lab Invest.

[43] Dodington DW, Lagree A, Tabbarah S, Mohebpour M, Sadeghi-Naini A, Tran WT (2021). Analysis of tumor nuclear features using artificial intelligence to predict response to neoadjuvant chemotherapy in high-risk breast cancer patients. Breast Cancer Res Treat.

[44] Kather JN, Krisam J, Charoentong P, Luedde T, Herpel E, Weis CA (2019). Predicting survival from colorectal cancer histology slides using deep learning: a retrospective multicenter study. PLoS Med.

[45] Beck AH, Sangoi AR, Leung S, Marinelli RJ, Nielsen TO, van de Vijver MJ (2011). Systematic analysis of breast cancer morphology uncovers stromal features associated with survival. Sci Transl Med.

[46] Shamai G, Binenbaum Y, Slossberg R, Duek I, Gil Z, Kimmel R (2019). Artificial intelligence algorithms to assess hormonal status from tissue microarrays in patients with breast cancer. JAMA Netw Open.

