# Supplementary material for: Deep learning-based predictive biomarker of pathological complete response to neoadjuvant chemotherapy from histological images in breast cancer
Source: J Transl Med. 2021 Aug 16;19:348. doi: 10.1186/s12967-021-03020-z (PMC8365907; doi:10.1186/s12967-021-03020-z)
Supplement: Supplementary file 1 — Additional file 1: Figure S1. The workflow of patient selection. Figure S2. Learning and inference processes of CNN I for TE identification. Figure S3. ROC curve and confusion matrices of CNN I for identifying TE. Figure S4. Learning and inference processes of CNN II for pCR prediction. Figure S5. ROC curves of CNN II for pCR prediction at tile-level. Figure S6. ROC curves of CNN II for pCR prediction based on TE on tile-level and WSI-level among subtypes in validation. Figure S7. Distributions of the pCR-score in the pCR group and the non-pCR group of the validation dataset. Table S1. Detailed NAC regimens of patients. Table S2. Demographic comparison between the primary and validation datasets. Table S3. Performance metrics of CNN I including accuracy, F1 score, AUC, sensitivity (recall), PPV (precision), specificity, and NPV. Table S4. Performance metrics including sensitivity (recall), PPV (precision), specificity, and NPV for biomarker-based models (T stage, nuclear grade, and Ki67). [file 12967_2021_3020_MOESM1_ESM.docx]

**Additional file 1**

Fig. S1 The workflow of patient selection.

*Abbreviations*: BC, breast cancer; NAC, neoadjuvant chemotherapy; N, number; HER2, human epidermal growth factor receptor 2; H&E, hematoxylin and eosin.

**
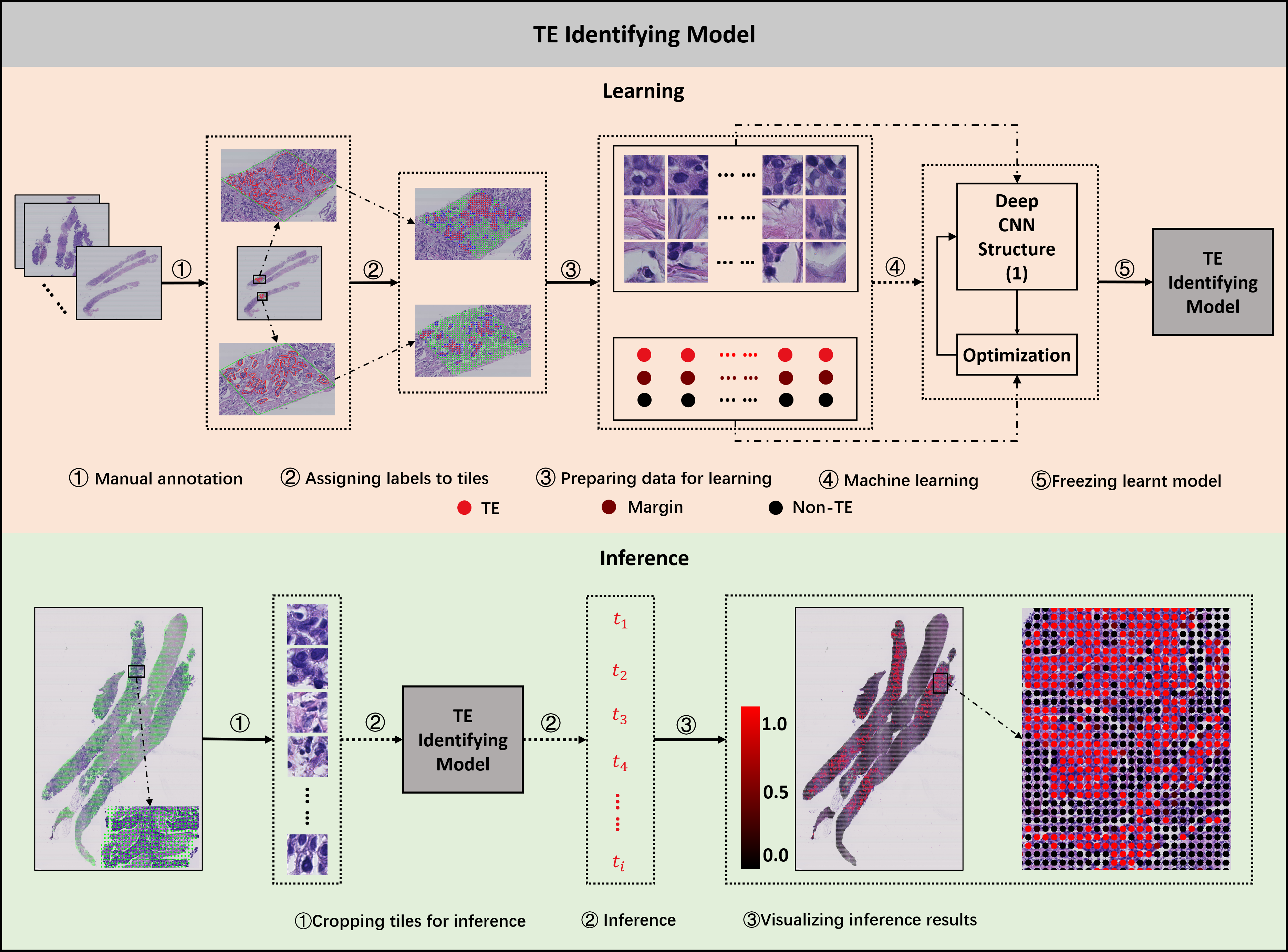
**

Fig. S2 Learning and inference processes of CNN Ⅰ for TE identification. Five steps in the learning process: ① tumor epithelium (TE) was manually annotated in a few representative tumor regions, ② labels of TE, non-TE, and Margin were assigned to each tiles which have been cropped at 128 × 128 pixels, ③ tiles and corresponding labels was prepared for learning, ④ machine learning process (optimization of the deep CNN structure) for TE identification, ⑤ the CNN Ⅰ was obtained by freezing the parameters of the optimized deep CNN architecture. Three steps in the inference process: ① the primary tissue areas of the input WSI was cropped into tiles of 128 × 128 pixels, ② tiles were input into CNN Ⅰ and mapped into corresponding probabilities of TE, ③ the predicted probabilities of TE were visualized on corresponding tiles.

Fig. S3 Performance of CNN Ⅰ for identifying TE. A. ROC curve of CNN Ⅰ for identifying TE. B. Confusion matrices of CNN Ⅰ for identifying TE at cutoff points of 0.5 (a) and 0.9999 (b).


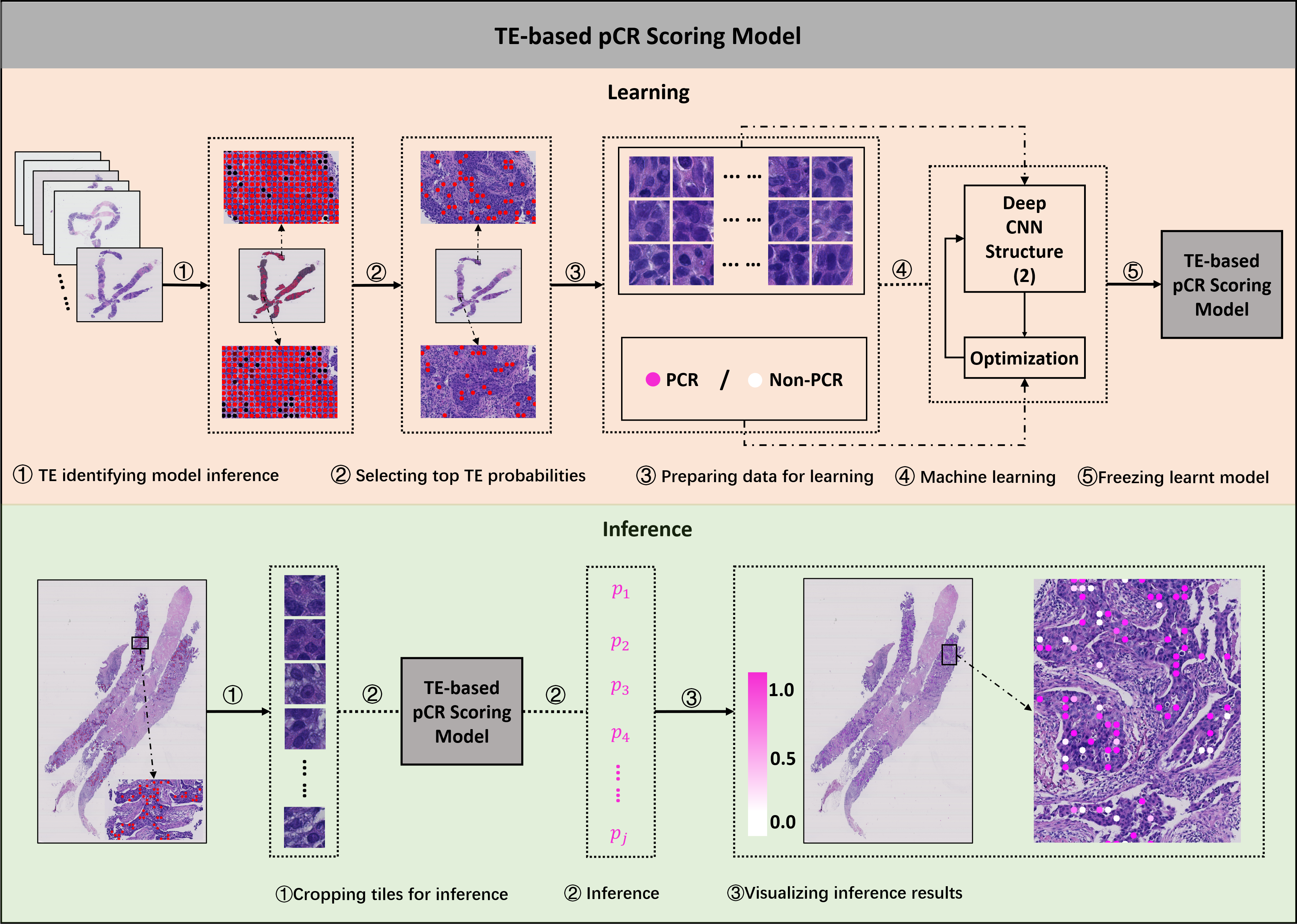


Fig. S4 Learning and inference processes of CNN Ⅱ for pCR prediction. Five steps in the learning process: ① tumor epithelium (TE) of a WSI was automatically identified by CNN Ⅰ, ② Tiles with high probabilities of TE were selected, ③ TE tiles with lables of pCR or non-pCR were settled for learning, ④ machine learning process (optimization of the deep CNN structure) for pCR predictions, ⑤ the CNN Ⅱ was obtained by freezing the parameters of the optimized deep CNN architecture. Three steps in the inference process: ① tiles of 128 × 128 pixels which have been identified by CNN Ⅰ with high probabilites of being TE were cropped, ② cropped tiles were input into CNN Ⅱ and mapped into probabilities of pCR, ③ the predicted probabilities of pCR were visualized on corresponding tiles.

Fig. S5 ROC curves of raw pCR-scores based on CNN Ⅱ for pCR prediction at tile-level. A. ROC curve of CNN Ⅱ on tile-level in the primary dataset. B. ROC curve of CNN Ⅱ on tile-level in the validation dataset.

Fig. S6 ROC curves of CNN Ⅱ for pCR prediction on tile-level and WSI-level among subtypes in validation. A. ROC curve of CNN Ⅱ on tile-level among subtypes in validation. B. ROC curve of CNN Ⅱ on WSI-level among subtypes in validation.

Fig. S7 Distributions of pCR-score in the pCR group and the non-pCR group in the validation dataset. The pCR group had a mean pCR-score of 0.342 (95% CI 0.227-0.457) while the non-pCR group had a mean pCR-score of 0.097 (95% CI 0.072-0.122), which showed that there was an obvious gap between their confidence intervals (no overlap) and the pCR-score in the pCR group was significantly higher than that in the non-pCR group (P<0.001).

**Table S1 Detailed NAC regimens of patients**

| Regimens | Four cycles | Six cycles | Eight cycles | Total |
| --- | --- | --- | --- | --- |
| Taxane-based | 58 | 46 | 13 | 117 |
| Anthrancycline-based | 12 | 3 | 1 | 16 |
| Anthrancycline- and Taxane-based | 226 | 100 | 81 | 407 |

**Table S2 Demographic comparison between the primary and validation datasets**

| Factors | Primary Dataset  (n,%) | Validation Dataset  (n,%) | *p value* |
| --- | --- | --- | --- |
| Age at diagnosis |  |  | 0.512 |
| < 50 | 255(58.9) | 59(55.1) |  |
| ≥ 50 | 178(41.3) | 48(44.9) |  |
| Menopausal status |  |  | 0.270 |
| Premenopausal | 266(61.4) | 59(55.1) |  |
| Postmenopausal | 167(38.6) | 48(44.9) |  |
| T stage |  |  | 0.914 |
| T1-T2  T2  T3  T4 | 200(46.2) | 50(46.7) |  |
| T3-T4  T3  T4 | 233(53.8) | 57(53.3) |  |
| N Stage |  |  | 0.914 |
| N0 | 40(52.9) | 5(52.3) |  |
| N1-N3 | 393(47.1) | 102(47.6) |  |
| ER Status |  |  | 0.303 |
| Positive | 269(62.1) | 70(65.4) |  |
| Negative | 164(37.9) | 37(34.6) |  |
| PR status |  |  | 0.364 |
| Positive | 258(59.2) | 66(61.7) |  |
| Negative | 175(40.8) | 41(38.3) |  |
| HER2 status |  |  | 0.433 |
| Positive | 119(27.5) | 32(29.9) |  |
| Negative | 314(72.5) | 75(70.1) |  |
| Subtypes |  |  | 0.636 |
| HR+/HER2- | 239(55.2) | 62(57.9) |  |
| HR+/HER2+ | 60(13.9) | 16(15.0) |  |
| HR-/HER2- | 75(17.3) | 13(12.1) |  |
| HR-/HER2+ | 59(13.6) | 16(15.0) |  |
| Ki67 Status |  |  | 1.000 |
| ≤ 20% | 61(14.1) | 17(15.90) |  |
| > 20% | 372(85.9) | 90(84.1) |  |
| Nuclear grade |  |  | 0.731 |
| 1 or 2 | 292(67.4) | 70(65.4) |  |
| 3 | 141(32.6) | 37(34.6) |  |
| sTILs(%) |  |  | 0.485 |
| Low | 238(55.0) | 61(57.0) |  |
| Moderate | 180(41.6) | 40(37.4) |  |
| High | 15(3.4) | 6(5.6) |  |
| Necrosis |  |  | 0.589 |
| No  0o | 344(79.4) | 88(82.2) |  |
| Yes | 89(20.6) | 19(17.8) |  |
| pCR |  |  | 0.891 |
| No | 352(81.3) | 86(80.4) |  |
| Yes | 81(18.7) | 21(19.6) |  |
| Total | 433(80.1) | 107(19.8) | - |

Bold indicates statistical significance (*P* < 0.05)

**Table S3 Performance matrics of CNN Ⅰ for identifying TE**

| Cutoff | F1 score | Accuracy | Sensitivity | PPV | Specificity | NPV |
| --- | --- | --- | --- | --- | --- | --- |
| 0.5 | 0.615 | 0.879 | 0.636 | 0.596 | 0.923 | 0.934 |
| 0.9999 | 0.446 | 0.884 | 0.306 | 0.823 | 0.988 | 0.888 |

*Note:* PPV is equal to precision, and sensitivity is equal to recall.

*Abrreviations:* PPV, positive predictive value; NPV, negative predictive value.

**Table S4 Performance metrics of biomarker-based models (T stage, nuclear grade, and Ki67)**

| Metrics | T stage-based ^a^ | | |  | Ki67-based ^a^ | | |  | NG-based ^a^ | | |  | Baseline 2 ^a^ | | |  | Integrated 2 ^c^ | | |
| --- | --- | --- | --- | --- | --- | --- | --- | --- | --- | --- | --- | --- | --- | --- | --- | --- | --- | --- | --- |
|  | Mean | 95% CI | *P* value |  | Mean | 95% CI | *P* value |  | Mean | 95% CI | *P* value |  | Mean | 95% CI | *P* value |  | Mean | 95% CI | *P* value |
| F1 score | 0 | - | - |  | 0 | - | - |  | 0 | - | - |  | 0.507 | 0.432-0.583 | 1.000 |  | 0.669 | 0.612-0.727 | **<0.001** |
| Accuracy | 0.801 | 0.775-0.827 | **<0.001** |  | 0.801 | 0.775-0.827 | **<0.001** |  | 0.801 | 0.775-0.827 | **<0.001** |  | 0.815 | 0.785-0.846 | 0.092 |  | 0.874 | 0.846-0.901 | **<0.001** |
| AUC | 0.453 | 0.389-0.507 | **<0.001** |  | 0.567 | 0.553-0.580 | **<0.001** |  | 0.604 | 0.573-0.635 | **<0.001** |  | 0.789 | 0.743-0.835 | 0.454 |  | 0.842 | 0.804-0.880 | **0.021** |
| Sensitivity | 0 | - | - |  | 0 | - | - |  | 0 | - | - |  | 0.494 | 0.400-0.587 | 0.388 |  | 0.660 | 0.556-0.763 | **<0.001** |
| PPV | 0 | - | - |  | 0 | - | - |  | 0 | - | - |  | 0.576 | 0.462-0.689 | **0.035** |  | 0.739 | 0.655-0.823 | **<0.001** |
| Specificity | 1 | - | - |  | 1 | - | - |  | 1 | - | - |  | 0.899 | 0.866-0.932 | **0.013** |  | 0.933 | 0.904-0.961 | 0.065 |
| NPV | 0.801 | - | - |  | 0.801 | - | - |  | 0.801 | - | - |  | 0.876 | 0.847-0/904 | 0.607 |  | 0.913 | 0.881-0.945 | **<0.001** |
| TP | 0 | - | **<0.001** |  | 0 | - | **<0.001** |  | 0 | - | **<0.001** |  | 4.125 | 3.400-4.850 | 0.267 |  | 5.438 | 4.660-6.215 | **<0.001** |
| FN | 8.563 | 7.445-9.680 | **0.004** |  | 8.563 | 7.445-9.680 | **0.004** |  | 8.563 | 7.445-9.680 | **0.004** |  | 4.438 | 3.355-5.520 | 0.607 |  | 3.125 | 1.944-4.307 | **<0.001** |
| FP | 0 | - | **<0.001** |  | 0 | - | **<0.001** |  | 0 | - | **<0.001** |  | 3.500 | 2.333-4.667 | **0.003** |  | 2.313 | 1.345-3.280 | 0.065 |
| TN | 34.44 | 33.32-35.56 | 0.607 |  | 34.44 | 33.32-35.56 | 0.607 |  | 34.44 | 33.32-35.56 | 0.607 |  | 30.94 | 29.60-32.29 | **0.004** |  | 32.13 | 30.67-33.58 | 0.065 |

*Note:* ^a^ refers to the *P* value was from that the comparisons with pCRscore-based model; ^c^ solely refers to the *P* value was from the comparisons of baseline model 2 and integrated model 2 (Baseline 2: T stage+Ki67+NG+sTILs+subtype; Integrated 2: T stage+Ki67+NG+sTILs+subtype+pCR-score).

*Abbreviations:* CI, confidence interval; AUC, area under the curve; NG. nuclear grade.

Bold indicates statistical significance (*P*< 0.05)
